# Activity of Pyrazinamide against *Mycobacterium tuberculosis* at Neutral pH in PZA-S1 Minimal Medium

**DOI:** 10.3390/antibiotics10080909

**Published:** 2021-07-26

**Authors:** Wanliang Shi

**Affiliations:** PZA Innovation LLC, 2401 West Belvedere Avenue, Baltimore, MD 21215, USA; wanliangshi@pzainnovation.com

**Keywords:** pyrazinamide, susceptibility testing, *Mycobacterium tuberculosis*, neutral pH

## Abstract

Susceptibility testing of tuberculosis (TB) drugs on *Mycobacterium tuberculosis* is essential for the rapid detection of strains resistant to the drugs, providing the patient with effective treatment, and preventing the spread of drug-resistant TB strains. Pyrazinamide (PZA) is one of the first-line agents used for the treatment of TB. However, current phenotypic PZA susceptibility testing is unreliable due to its performance in acidic pH conditions. The aims of this study were to develop minimal media to determine the activity of PZA at a neutral pH at 37 °C to avoid problems caused by an acidic pH, which is currently used in PZA susceptibility tests, and to identify PZA-resistant *M. tuberculosis* in media with reproducibility and accuracy. Different minimal media were used to determine the activity of PZA using the broth microdilution method with *M. tuberculosis* H37Ra as the reference strain. The PZA-S1 minimal medium was proposed as the most suitable medium. PZA inhibited the growth of *M. tuberculosis* in PZA-S1 at a neutral pH of 6.8, which is the optimal pH for *M. tuberculosis* growth. Moreover, PZA showed activity at a neutral pH on a PZA-S1 agar plate when using the disk diffusion method. PZA-resistant *M. tuberculosis* could be identified at a neutral pH in PZA-S1 minimal medium. This study establishes valuable information regarding the testing of PZA’s susceptibility in relation to *M. tuberculosis* at a neutral pH of 6.8 with reliability and accuracy in clinical settings.

## 1. Introduction

Tuberculosis (TB) is the leading infectious disease worldwide and the pandemic could make it worse [1]. Ten million people fell ill from TB and 1.4 million died in 2019 [2]. Currently, TB treatment involves a 6-month regimen of a combination of four drugs: rifampicin (RIF), isoniazid (INH), pyrazinamide (PZA), and ethambutol (EMB). PZA is a cornerstone of TB treatment, and was introduced into TB chemotherapies in order to reduce the length of treatment from 9–12 months to 6 months. PZA is also playing a role in new regimens designed to further shorten the treatment duration and more effectively treat multidrug-resistant tuberculosis (MDR-TB) [3,4]. However, PZA is the least understood among the TB drugs. It has been known that PZA is active in an acidic environment (pH 5.0–5.5), but not at a neutral pH, in conventional culture media [5]. PZA is a prodrug that is converted to its active form, pyrazinoic acid (POA), by pyrazinamidase/nicotinamidase encoded by the *pncA* gene in *Mycobacterium tuberculosis* [6,7]. Several target proteins of PZA were suggested to inhibit fatty acid synthase (Fas I) [8], ribosomal protein S1 (RpsA) [9,10,11], aspartate decarboxylase (PanD) [12,13,14], caseinolytic protease (ClpC1) [15] and guanosine pentaphosphate synthetase (GpsI) [16,17]. However, there are controversies regarding the target proteins of PZA, Fas I [18], RpsA [19] and PanD [20].

Although PZA is very important, due to technical difficulties and frequent false results, many countries at high risk of TB do not routinely conduct PZA susceptibility testing. This notorious challenge was caused by the performance of PZA at an acidic pH during susceptibility testing [21,22,23]. False susceptibility results with PZA have commonly been caused by an acidic pH, in which at least 10% of clinical isolates fail to grow and false resistance results to PZA are also caused by a large inoculum size, which changes the acidic pH to a neutral pH rapidly due to bacterial growth [24,25]. The prevalence of PZA resistance was estimated to be 16.2% among all TB cases, 41.3% among patients at high risk of MDR-TB, and 60.5% among MDR-TB cases. The global burden is 1.4 million new PZA-resistant TB cases annually [26,27,28,29]. Resistance to any primary drugs (including PZA) makes the treatment of the disease more challenging and costlier. As such, reliable detection of these resistant strains is essential for effective TB patient management. Currently, The Global Tuberculosis Program of the World Health Organization (WHO) has the mandate to develop and disseminate evidence-based policies for TB prevention, diagnosis, treatment and care [30]. Therefore, in order to treat TB effectively and combat the spread of drug resistance, there is an urgent need for reliable PZA susceptibility testing to be performed routinely worldwide. Phenotypic drug susceptibility testing is the gold standard for determining *M. tuberculosis* susceptibility [31] and interpreting the results of molecular susceptibility tests. Thus, a reliable form of phenotypic PZA susceptibility testing is urgently needed.

In previous studies, the culture-based activity of PZA was tested in conventional culture media or only slightly modified based on the conventional media [5,32,33,34,35] and phosphate-buffered saline (PBS) [36,37]. Since the discovery of *M. tuberculosis*, the cultivation of mycobacteria has been intensively studied. *M. tuberculosis* is able to grow in simple salt medium [38,39]. The aims of this study were to develop minimal media to determine the activity of PZA at a neutral pH at 37 °C to avoid problems caused by an acidic pH, which is currently used in PZA susceptibility tests, and to identify PZA-resistant *M. tuberculosis* in the media with reproducibility and accuracy.

## 2. Results

### 2.1. Determination of PZA Activity at Neutral pH in Minimal Medium with Amino Acid as the Nitrogen Source

**Figure 1:**
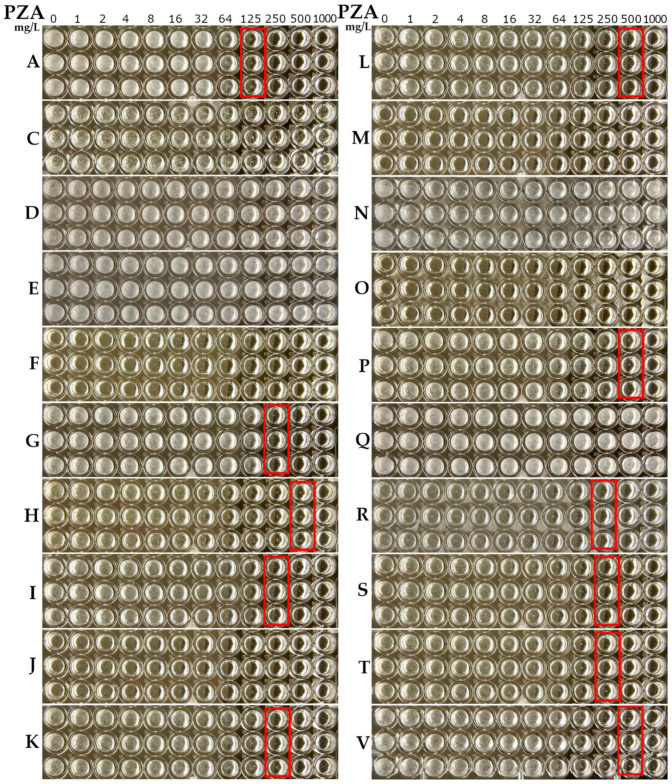
PZA susceptibility and growth of *M. tuberculosis* at pH 6.8 using the broth microdilution method in different minimal media and ingredients (described in Table 1) with an amino acid at 2 g/L: the L-Ala result in panel **A**; the L-Cys result in panel **C**; the L-Asp result in panel **D**; the L-Glu result in panel **E**; the L-Phe result in panel **F**; the Gly result in panel **G**; the L-His result in panel **H**; the L-Ile result in panel **I**; the L-Tyr result in panel **J**; the L-Lys result in panel **K**; the L-Leu result in panel **L**; the L-Met result in panel **M**; the L-Asn result in panel **N**; the L-Trp result in panel **O**; the L-Pro result in panel **P**; the L-Gln result in panel **Q**; the L-Arg result in panel **R**; the L-Ser result in panel **S**; the L-Thr result in panel **T**; the L-Val result in panel **V**. Red rectangular boxes indicate the lowest PZA concentration wells, which resulted in no visible *M. tuberculosis* growth.

*M.tuberculosis* was able to grow in simple media [38,39,40] and some amino acids were utilized as the primary nitrogen source for *M. tuberculosis* growth [41]. In Figure 1, the activity of PZA is presented, which was determined at a neutral pH of 6.8 in minimal media using amino acid as the nitrogen source. The ingredients of the media are listed in Table 1. The activity of PZA was observed as the growth inhibition of *M. tuberculosis* occurring in the presence of PZA and its visible growth in the absence of PZA in the medium. The results for the activity of PZA at a neutral pH in the minimal media (shown in Figure 1) were divided to three groups. In Group I, the minimal inhibitory concentration (MIC) of PZA against *M. tuberculosis* was greater than 1000 mg/L and one of five kinds of amino acids, either L-Aspartic acid (L-Asp) (Figure 1D), L-Glutamic acid (L-Glu) (Figure 1E), L-Asparagine (L-Asn) (Figure 1N), L-Glutamine (L-Gln) (Figure 1Q), or L-Cysteine (L-Cys) (Figure 1C), was included as the nitrogen source in the minimal media. In Group II, the MIC of PZA was between 125 mg/L to 500 mg/L, with one of eleven kinds of amino acids, either L-Histidine (L-His) (Figure 1H), L-Proline (L-Pro) (Figure 1P), L-Valine (L-Val) (Figure 1V), L-Leucine (L-Leu) (Figure 1L), L-Arginine (L-Arg) (Figure 1R), L-Lysine (L-Lys) (Figure 1K), or L-Serine (L-Ser) (Figure 1S), L-Threonine (L-Thr) (Figure 1T), L-Isoleucine (L-Ile) (Figure 1I), glycine (Gly) (Figure 1G), or L-Alanine (L-Ala) (Figure 1A), included as the nitrogen source. The lowest MIC of PZA (125 mg/L) was found in the medium with L-Ala. The MIC of PZA reached 250 mg/L in the medium with the following amino acids: L-Arg, or L-Lys, or L-Ser, or L-Thr, or L-Ile, or Gly and 500 mg/L with the following amino acids: L-His, or L-Pro, or L-Val, or L-Leu. In Group III, the MIC of PZA was not detectable since *M. tuberculosis* failed to grow in the minimal media, which included either L-Methionine (L-Met) (Figure 1M), L-Phenylalanine (L-Phe) (Figure 1F), L-Tryptophan (L-Trp) (Figure 1O) or L-Tyrosine (L-Tyr) (Figure 1J) as the nitrogen source.

The status of *M. tuberculosis* growth is shown in the first column (on the left side of each sample) in Figure 1 and is summarized in Table 1. There was luxuriant growth (+++) in the media with either L-Asn, L-Asp, L-Gln, L-Glu, L-Val, Gly, or L-Ala amino acids. Moreover, there was visible or moderate growth in the media with either L-Cys, L-His, L-Pro, L-Leu, L-Arg, L-Lys, L-Ser, L-Thr, or L-Ile amino acids. The results of the growth state of *M. tuberculosis* using amino acid as the primary nitrogen source with glycerol as carbon source in the synthetic media were consistent with previous studies [3,42]. In conventional culture media, L-Asn or L-Asp are usually used as the nitrogen source [39,40,43,44]. The fact that PZA was not active at a neutral pH in conventional culture media is consistent with previous studies [3,45,46,47].

To evaluate whether the concentration of amino acid affects the activity of PZA in the minimal media, L-Asn and L-Ala were selected for the test. While the concentration of L-Asn was low in the minimal medium, at 0.1 g/L, the results presented in Table 1 show that PZA had no activity at a concentration of 1000 mg/L. In addition, in the medium with L-Ala as the nitrogen source, the MIC of PZA showed no changes when the concentration of L-Ala was in the range from 4 to 0.5 g/L at pH 6.8 in the minimal media (Table 1). The growth rate did not change significantly due to the change in the concentration of either L-Asn or L-Ala as the nitrogen source in the media. Since the growth rates between L-Asn and L-Ala (as the nitrogen sources in the media) were similar, this indicated that the activity of PZA was unlikely to be correlated with the state of growth of *M. tuberculosis* but, instead, with the type of nutrient in the culture media. In comparison, L-Ala was the best nitrogen source among the amino acids, with the lowest MIC of PZA and luxuriant growth in the study. The medium with 2 g/L of L-Ala and other ingredients listed in Table 1 is named PZA-S1 minimal medium in the remainder of the study.

In addition, the relation between the glycerol concentration and the activity of PZA in the PZA-S1 medium was evaluated. While the concentration of glycerol was 5 mL/L, 20 mL, or 40 mL/L in the PZA-S1 minimal media, the MIC of PZA was the same as that of 10 mL/L of glycerol (200 mg/L). The growth of *M. tuberculosis* was poorer with 40 mL/L of glycerol than with 10 mL/L of glycerol in PZA-S1 media. Moreover, this led to an *M. tuberculosis* growth defect when MgSO_4_ was replaced with CaCl_2_ at the same concentration in the PZA-S1 medium.

### 2.2. Effect of Activity of PZA by pH in PZA-S1 Minimal Medium

The effect of pH on the activity of PZA in conventional culture media has been known for a long time [5]. To identify whether pH has an effect on PZA susceptibility in PZA-S1 minimal medium, the PZA MIC of *M. tuberculosis* was tested in PZA-S1 minimal media in the pH range from 5.0 to 8.5. It is shown by the results in Table 2 that the activity of PZA was significantly affected by pH in the PZA-S1 minimal media at 37 °C over 2 weeks of incubation. The MIC of PZA to *M. tuberculosis* was 100 mg/L at pH 8.5, and 200 µg/mL at pH 6.5 to 8.0. At pH 6.0 and 5.5, the MICs of PZA to *M. tuberculosis* were 100 and 25 mg/L, respectively. There was no growth at pH 5.0. As such, it is clear that the MIC of PZA is affected by pH. The value of the MIC of PZA at a neutral pH was eight-fold that of the MIC at pH 5.5 in PZA-S1 media.

### 2.3. Identification of PZA-Resistant M. tuberculosis at Neutral pH 6.8 in PZA-S1 Minimal Medium

**Figure 2:**
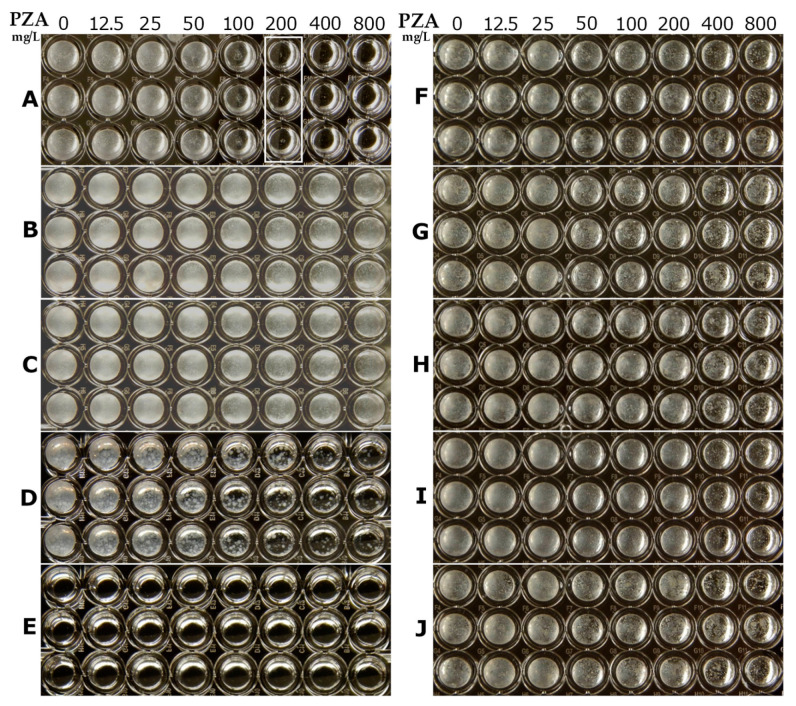
Determination of MIC of PZA against different *M. tuberculosis* strains using the broth microdilution method. (**A**), *M. tuberculosis* H37Ra, PZA-susceptible strain at pH 6.8 in PZA-S1 minimal medium; (**B**), *M. tuberculosis* H37Ra, PZA-susceptible strain at pH 6.8 in Sauton’s medium; (**C**), *M. tuberculosis* H37Ra, PZA-susceptible strain at pH 6.8 in Middlebrook 7H9 medium; (**D**), *M. tuberculosis* H37Ra, PZA-susceptible strain at pH 5.9 in Middlebrook 7H9 medium; (**E**), *M. tuberculosis* H37Ra, PZA-susceptible strain at pH 5.5 in Middlebrook 7H9 medium; (**F**), *M. tuberculosis* H37Ra PZA^R^ #6 strain (PncA mutation L159P) at pH 6.8 in PZA-S1 minimal medium; (**G**), *M. tuberculosis* H37Ra PZA^R^ #78 strain (PncA mutation C138R) at pH 6.8 in PZA-S1 minimal medium; (**H**), *M. tuberculosis* H37Ra PZA^R^ #98 strain (PncA mutation W68R) at pH 6.8 in PZA-S1 minimal medium; (**I**), *M. tuberculosis* H37Ra PZA^R^ #106 strain (PncA mutation D49G) at pH 6.8 in PZA-S1 minimal medium; (**J**), *M. tuberculosis* H37Ra PZA^R^ #186 strain (PncA mutation G132A) at pH 6.8 in PZA-S1 minimal medium.

The broth microdilution method allows for the susceptibility testing of multiple drugs in multiple concentrations. It is easy to perform and does not require special equipment or expensive supplies [48]. The results of the standard broth microdilution method were obtained over 3 weeks at 37 °C with inocula at 1 to 100 dilution from a 0.5 McFarland unit of *M. tuberculosis*. In this study, the MIC of the PZA-susceptible strain, *M. tuberculosis* H37Ra, was 200 mg/L at pH 6.8 in the PZA-S1 minimal medium over two weeks of incubation (Figure 2A, labeled with a white box). In contrast, *M. tuberculosis* H37Ra was grown at a neutral pH of 6.8 with PZA at a concentration of 800 mg/L in conventional culture media, such as Sauton’s medium and Middlebrook 7H9 (Figure 2B,C). Although PZA slightly inhibited the growth of *M. tuberculosis* at an acidic pH of 5.9 in Middlebrook 7H9 medium when the concentration of PZA was greater than 100 mg/L (Figure 2D), the degree of inhibition of PZA at 200 mg/L against *M. tuberculosis* at a neutral pH of 6.8 in the PZA-S1 medium led to no visible growth. There was a defect in the growth of *M. tuberculosis* with 1 to 100 dilution from a 0.5 McFarland unit of *M. tuberculosis* at an acidic pH of 5.5 in Middlebrook 7H9 medium (Figure 2E). The five PZA-resistant *M. tuberculosis* strains containing a mutation in the *pncA* gene showed no inhibition of growth at PZA concentrations up to 800 mg/L (Figure 2F–J). Thus, the MIC of PZA was 200 mg/L for *M. tuberculosis* H37Ra and greater than 800 mg/L for the five PZA-resistant strains. The standard broth microdilution method could therefore be used to identify PZA resistance in *M. tuberculosis* at neutral pH at 37 °C in PZA-S1 media.

### 2.4. Susceptibility Testing of First-Line TB Drugs and Determination of pH during Growth of M. tuberculosis at Neutral pH 6.8 in PZA-S1 Minimal Medium

**Figure 3:**
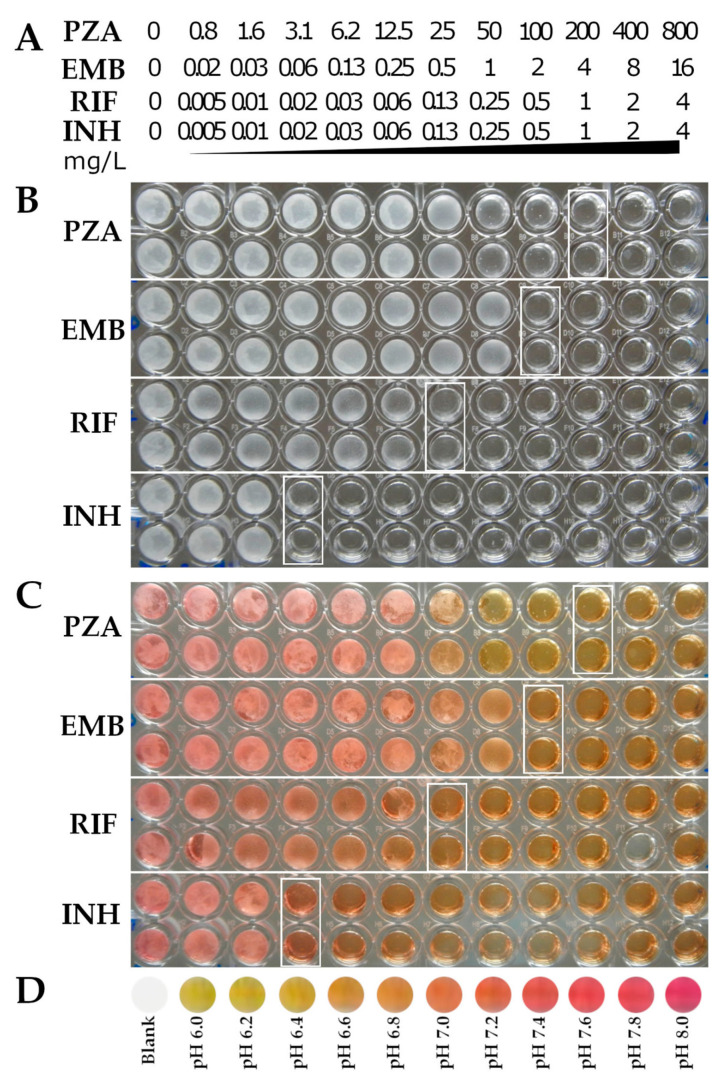
Determination of MICs of first-line TB drugs against *M. tuberculosis* and pH at 37 °C over 2 weeks at pH 6.8 in PZA-S1 minimal medium. (**A**), The concentrations of first-line TB drugs in the 96-well plate from wells 1 to 12 (PZA from 0 to 800 mg/L, EMB from 0 to 16 mg/L, RIF and INH from 0 to 4 mg/L). (**B**), No growth, labeled with white rectangular boxes on the microdilution plate for the first-line TB drug susceptibility tests in *M. tuberculosis*. (**C**), No growth, labeled with white rectangular boxes on the microdilution plate for the first-line TB drug susceptibility tests in *M. tuberculosis* and medium with phenol red at 15 mg/L. (**D**), Color standard of pH 6.0 to 8.0 with phenol red in PZA-S1 medium and blank control.

To further evaluate the application of the PZA-S1 minimal medium in determining the susceptibility of first-line TB drugs, susceptibility tests of *M. tuberculosis* H37Ra were carried out using the broth microdilution method. The concentrations of drugs in the 96-well plate were labeled as shown in Figure 3A. Each test on each drug sample was performed in duplicate. The MICs of PZA, EMB, RIF and INH were 200 mg/L, 2 mg/L, 0.13 mg/L and 0.02 mg/L, respectively (Figure 3B). The MICs of EMB, RIF and INH were found to be in the normal range via the broth microdilution method, as found in a previous study [49]. The MICs of PZA are not available from any previous susceptibility tests at neutral pH.

Phenol red is a pH indicator in culture medium that exhibits a gradual transition from yellow to red over a pH range of 6.2 to 8.2 [50]. In order to determine how the pH of *M. tuberculosis* changes during growth in the PZA-S1 minimal medium, phenol red was added at a concentration of 15 mg/L in the culture of *M. tuberculosis* as the pH of the culture can be determined by its color. Regardless of PZA or other first-line TB drugs in the culture, the pH at which the drug completely inhibited the growth of *M. tuberculosis* in the well was ~6.8 (Figure 3C, right side of white box) at 37 °C over 2 weeks of incubation. The pH was ~7.0 in the luxuriant growth wells of *M. tuberculosis* (Figure 3C) compared with the color standard indicated by phenol red (Figure 3D). In addition, the results of the pH of *M. tuberculosis* H37Ra culture were measured at different time points, as shown in Table 3. The pH of *M. tuberculosis* culture increased slightly from 6.8 to 7.2 at 37 °C over 18 days of incubation and then decreased to 6.5 over 30 days of incubation in the PZA-S1 minimal medium. In contrast, there was no significant change in the control group (Table 3). Therefore, the activity of PZA against *M. tuberculosis* occurred at a neutral pH at 37 °C throughout the cultivation.

### 2.5. Activity of PZA at Neutral pH on PZA-S1 Agar Plate

**Figure 4:**
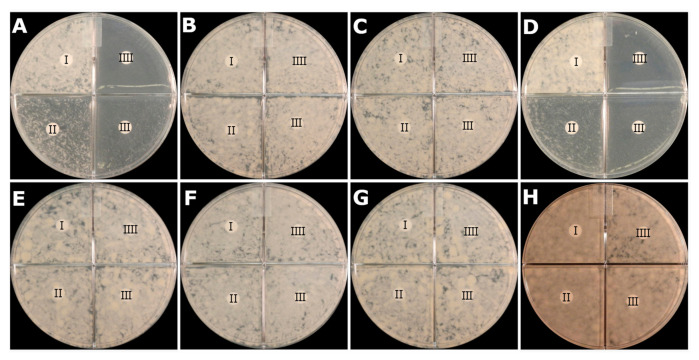
Growth inhibition of *M. tuberculosis* by PZA at pH 6.8 on PZA-S1 minimal medium agar plate. (**A**), *M. tuberculosis* H37Ra, PZA-susceptible strain; (**B**), *M. tuberculosis* H37Ra PZA^R^ #6 strain (PncA mutation L159P); (**C**), *M. tuberculosis* H37Ra PZA^R^ #78 strain (PncA mutation C138R); (**D**), *M. tuberculosis* H37Ra NR-122, PZA-susceptible strain; (**E**), *M. tuberculosis* H37Ra PZA^R^ #98 strain (PncA mutation W68R); (**F**), *M. tuberculosis* H37Ra PZA^R^ #106 strain (PncA mutation D49G); (**G**), *M. tuberculosis* H37Ra PZA^R^ #186 strain (PncA mutation G132A). (**H**), *M. tuberculosis* H37Ra on 7H11 agar plate. paper disc I, 6 mm, DMSO control; paper disc II, 6 mm paper disc containing 400 µg of PZA; paper disc III, 6 mm paper disc containing 800 µg of PZA; paper disc IIII, 6 mm paper disc containing 1600 µg of PZA.

To detect whether PZA is active against *M. tuberculosis* at a neutral pH of 6.8 on an agar plate, a test was performed on a PZA-S1 medium agar plate using the disk diffusion method. The growth characteristics of *M. tuberculosis* in broth and on agar plates are different. Agar contains long-chain fatty acids and lipids, which have a strong inhibitory effect on the growth of *M. tuberculosis* [51]. Indeed, the growth of *M. tuberculosis* H37Ra displayed a severe defect on the PZA-S1 minimal medium agar plate without supplements. This phenomenon has been described in a previous study, in which albumin was able to eliminate the inhibitory effect on growth on an agar plate [43]. when albumin, at a concentration of 0.5 g/L, was supplemented on the PZA-S1 minimal medium agar plate, there was a luxuriant growth of *M. tuberculosis* on the plate (Figure 4, all quadrant I parts without PZA control). Moreover, there was an inhibition of growth of PZA-susceptible *M. tuberculosis* H37Ra strains when a paper disc containing 400 µg, 800 µg or 1600 µg of PZA was placed on the PZA-S1 media agar plate (Figure 4A,D: quadrants II, III and IIII), but no inhibition on the other five PZA-resistant strains with the paper disc containing PZA (Figure 4B–G, quadrants II, III and IIII). Furthermore, PZA did not inhibit the growth of *M. tuberculosis* H37Ra when the paper disc contained 400 µg, 800 µg or 1600 µg of PZA on the conventional medium, 7H11 agar plate (Figure 4H). There was no clear circular zone for *M. tuberculosis* because of even diffusion throughout the long incubation time, which was also described in the previous literature [52], but it is very clear that there was no growth of PZA-susceptible strains in the quadrant with the PZA-containing paper disc at pH 6.8 on the PZA-S1 agar plate; however, this was not the case for PZA-resistant strains containing a mutation in the *pncA* gene. For the first time, these results clearly demonstrate the activity of PZA against *M. tuberculosis* at a neutral pH at 37 °C on an agar plate.

## 3. Discussion

PZA, despite its sterilizing activity in vivo, has no activity against *M. tuberculosis* at neutral pH in conventional culture media, but it does present activity at an acidic pH. This is a distinctly unusual phenomenon among the TB drugs [5]. In this study, the activity of PZA against *M. tuberculosis* was investigated at a neutral pH in non-conventional culture media at 37 °C by the standard broth microdilution method. We demonstrated, for the first time, that PZA actively inhibits the visible growth of *M. tuberculosis* at a neutral pH of 6.8 at 37 °C in PZA-S1 minimal medium and on an agar plate. The MIC of PZA was determined at 200 mg/L for *M. tuberculosis* H37Ra at a neutral pH of 6.8 at 37 °C using the broth microdilution method in PZA-S1 minimal medium. Moreover, PZA was found to inhibit the growth of *M. tuberculosis* H37Ra at a neutral pH of 6.8 at 37 °C on a PZA-S1 agar plate via the disk diffusion method. Furthermore, PZA-resistant strains with a *pncA* mutation were identified in PZA-S1 broth or on a PZA-S1 agar plate at a neutral pH at 37 °C. It has been reported that PZA is active against *M. tuberculosis* H37Ra and two clinical isolates at a neutral pH at 28 °C by the determination of the microcolony-based growth rate [34]. PZA also displayed activity against *M. tuberculosis* H37Ra at a neutral pH of 7.0 at 37 °C in PBS via colony-forming unit (CFU) counting [36]. In previous studies, *M. tuberculosis* subjected to anaerobic or microaerobic conditions [53], nutrient starvation [36,37], a low incubation temperature [34,54], inhibitors of the *M. tuberculosis* respiratory machinery and compounds that disrupt membrane potential and energetics [35,53,55,56], the efflux pump inhibitor reserpine [24,57], ultraviolet light and some weak acids [37,56] was more susceptible to PZA. Therefore, the activity of PZA varies in different environments and should be detectable at a neutral pH at 37 °C in a minimal medium, such as PZA-S1.

*M. tuberculosis* has a flexible metabolic pathway and utilizes a diversity of nutrients [39,42,43,58]. *M. tuberculosis* utilizes multiple amino acids as nitrogen sources in vivo and in vitro [41,59,60,61]. In this study, the activity of PZA was determined at a neutral pH of 6.8 in a minimal medium with each of twenty L-amino acids as the primary nitrogen source. Interestingly, *M. tuberculosis* H37Ra was susceptible to PZA in our minimal media with either one of nine kinds of amino acids as the primary nitrogen source in this study. PZA did not show activity at a neutral pH in the minimal media with amino acids that are commonly used in conventional culture media, such as L-Asn, L-Asp, L-Glu or L-Gln. It was believed that L-Asn, L-Glu, and L-Asp served as good stimulants for the growth of *M. tuberculosis*, whereas L-Ala, L-His, and L-Pro possibly stimulated growth to a lesser degree [43,60,62]. For example, *M. tuberculosis* and other mycobacteria displayed luxuriant growth when L-Ala was utilized as the primary nitrogen source in synthetic media [39,41]. In this study, the growth of *M. tuberculosis* was similar in the minimal medium with either L-Ala or L-Asn as the primary nitrogen source. However, PZA was active in the medium with L-Ala, but not in the medium with L-Asn. The MIC of PZA does not change when the concentration of L-Ala or L-Asn varies in the medium. In previous studies, it was thought that PZA only acts on no-growing *M. tuberculosis* [63,64,65]. In this study, PZA clearly inhibited the growth of *M. tuberculosis* H37Ra at pH 6.8 in the PZA-S1 medium. In addition, PZA displayed activity in the minimal medium with Gly, or L-Val, or L-Ser, or L-Arg, or L-Thr, or L-Lys, or L-Cys, or L-Pro, or L-His, or L-Leu, or L-Ile as the primary nitrogen source. The MIC of PZA varied with different amino acids as the nitrogen source in the minimal media. Therefore, it is reasonable to suggest that PZA activity is related to the specific nitrogen source in the minimal media. PZA can act on growing *M. tuberculosis* at a neutral pH in minimal media. The susceptibility of PZA is likely related to the different kind of amino acid as nitrogen sources in the minimal medium. As for the relationship between PZA activity and other nutrient elements, such as carbon sources, salts and trace elements, it is worthy of further research. Likewise, the data demonstrate that PZA activity, apart from at an acidic pH, could be useful for understanding the mechanism of action of PZA at a neutral pH and should therefore be considered for PZA susceptibility testing.

pH is a very important factor in the activity of PZA [5]. There is no doubt that there is an acidic pH in the phagosome of macrophages [66] and in TB lesions [67]. In general, it is believed that inflammation caused by mycobacteria infections leads to an acidic environment [57]. However, the mechanism of the acidic environment formation in TB lesions is unclear. Moreover, the pH was dynamic during *M. tuberculosis* growth and the direction of the pH, toward acidity or alkalinity, depends on the media [68,69]. In the PZA-S1 minimal medium, the pH changed slightly during the growth of *M. tuberculosis*, but it remained in the neutral pH range (from 6.5 to 7.2). Therefore, the level of acidity or alkalinity during the growth of *M. tuberculosis* was unlikely to be related to the PZA activity in the PZA-S1 medium but, instead, to the nitrogen source itself.

PZA is an important drug, for which it is notoriously difficult to perform susceptibility testing and to interpret the susceptibility test results, partly due to the fact that testing occurs at an acidic pH, which inhibits growth of *M. tuberculosis* [21,65,70,71]. Only two broth culture-based methods are currently FDA approved for PZA susceptibility testing: the VersaTREK MYCO TB system and the BACTEC MGIT 960 [21]. Both of these methods are performed at pH 5.9 in 7H9 media. However, the critical concentration of PZA is 100 mg/L in BACTEC MGIT 960 and 300 mg/L in the VersaTREK MYCO TB test [21]. The critical concentration is defined as the lowest concentration of an anti-TB agent in vitro that will inhibit the growth of 99% of phenotypically wild type strains of *M. tuberculosis* complex, but 90% inhibition was defined for PZA, which is lower than the standard [72]. The BACTEC MGIT 960 broth culture method is the only WHO-recommended method for PZA susceptibility testing, even though it is reportedly associated with a high rate of false-positive resistance results [72]. Due to those reasons, PZA susceptibility testing is often not conducted and is rarely used to inform treatment. The increasing threat to TB therapy posed by PZA-resistant strains of *M. tuberculosis* necessitates a reliable PZA susceptibility testing method [22,34]. To solve the problem caused by an acidic pH in PZA susceptibility testing, researchers developed PZA susceptibility tests at a neutral pH. However, these methods have not been widely used [34,73]. In this study, we demonstrated that PZA susceptibility testing can be performed in PZA-S1 medium using the standard broth microdilution method, along with other first-line TB drugs (Figure 3). The values of the MICs of RIF, INH and EMB in PZA-S1 broth were in the susceptible range compared with the results achieved using a Sensititre MYCOTB MIC plate [49,74] and a UKMYC5 plate [75]. PZA is not included in the Sensititre MYCOTB and UKMYC5 plates. Moreover, in the PZA-S1 medium, the susceptibility of all of the first-line TB drugs can be tested together and the results can be obtained at the same time. The MIC values of PZA were consistent regardless of the inoculum size, ranging from 1 to 200 and 1 to 50 dilutions of the standard inoculum size (a 0.5 McFarland unit) in our PZA susceptibility tests. It is, therefore, likely to be used in clinical testing.

In diagnostic laboratories, the disk diffusion test is widely used to determine the susceptibility of bacteria to different antibiotics on an agar plate. An effective drug will produce a zone of inhibition, while there may not be a clear zone for a resistant strain [76]. Due to the long incubation time for the growth of *M. tuberculosis*, there is no clear zone in susceptibility tests of TB drugs [52,77]. PZA has not presented any inhibition to the growth of *M. tuberculosis* on a conventional culture media agar plate before. However, in this study, it was clearly demonstrated that PZA could inhibit the growth of *M. tuberculosis* on an agar plate. There is therefore the potential of developing an agar proportion method to determine the susceptibility of *M. tuberculosis* to PZA.

In this study, *M. tuberculosis* H37Ra was used as the reference strain since it has the same susceptibility to most TB drugs, including PZA, as that of *M. tuberculosis* H37Rv and its application is convenient in BSL2 laboratories [78]. Although two *M. tuberculosis* H37Ra and five PZA-resistant strains were identified as being susceptible to PZA at a neutral pH of 6.8 in PZA-S1 broth and on an agar plate at 37 °C, the testing must be verified with *M. tuberculosis* H37Rv and clinical isolates in a future study.

## 4. Materials and Methods

### 4.1. Culture Media

The Middlebrook 7H9 broth and 7H11 agar were from Becton, Dickinson and Company (Difco 271310 and Difco 283810, BD, Sparks, MD, USA). Other minimal media were prepared in the laboratory. The minimal media contained: 2 g/L amino acid, 0.5 g/L KH_2_PO_4_ (P5656, Sigma-Aldrich, Co., St. Louis, MO, USA), 0.5 g/L MgSO_4_ (M2643, Sigma-Aldrich, Co., St. Louis, MO, USA), 0.5 g/L citric acid (C7129, Sigma-Aldrich, Co., St. Louis, MO, USA), 10 mL/L glycerol (G33, Fisher Chemical, Fair Lawn, NJ, USA), 30 mg/L ferric ammonium citrate (F5879, Sigma-Aldrich, Co., St. Louis, MO, USA), 0.5 mg/L biotin (B4639, Sigma-Aldrich, Co., St. Louis, MO, USA), 1 mg/L pyridoxine hydrochloride (P6280, Sigma-Aldrich, Co., St. Louis, MO, USA) and 0.5 mg/L ZnSO_4_ (Z0251, Sigma-Aldrich, Co., St. Louis, MO, USA). The types of amino acid were included either L-Asp, L- Glu, L-Asn, L-Gln, L-Cys, L-His, L-Pro, L-Val, L-Leu, L-Arg, L-Lys, L-Ser, L-Thr, L-Ile, Gly, L-Ala, L-Met, L-Phe, L-Trp, or L-Tyr (Sigma-Aldrich, Co., St. Louis, MO, USA). According to the needs of the experiment, the concentration of L-Asn, L-Ala or glycerol was adjusted in the minimal medium. For example, L-Asn was used at concentrations of 0.1 g/L, 0.5 g/L, or 2 g/L in the minimal media (Table 1). L-Ala was used at concentrations of 0.5 g/L, 1.0 g/L, 2.0 g/L, or 4.0 g/L in the minimal media (Table 1). Glycerol was used at concentrations of 5 mL/L, 10 mL/L, 20 mL/L or 40 mL/L in the minimal medium with 2 g/L L-Ala as the nitrogen source. The ingredients were dissolved in Milli-Q deionized type I ultrapure water. The pH of the media was adjusted with 1 M of sodium hydroxide (S8045, Sigma-Aldrich, Co., St. Louis, MO, USA) and sterilized by filtering through a 0.2 µm membrane filter (Catalog # 431224, Corning, NY, USA). All media were freshly prepared.

### 4.2. Bacterial Strain and Culture Conditions

*M. tuberculosis* H37Ra strains were used as the reference strains, with one being obtained from BEI Resources (ATCC, beiresource.org) and the other being a gift from Anthony D. Baughn, University of Minnesota Medical School (Table 4). The strains were grown on Middlebrook 7H11 agar plates supplemented with 0.5% (*v*/*v*) glycerol and 10% (*v*/*v*) albumin dextrose catalase (ADC) (Fisher Scientific, Fair Lawn, NJ, USA) for 3–4 weeks. PZA was purchased from Sigma-Aldrich, Co. (Catalog # P7136, Sigma-Aldrich, Co., St. Louis, MO, USA) and dissolved in dimethyl sulfoxide (DMSO) (D2650, Sigma-Aldrich Co., St. Louis, MO, USA). The stock solution of PZA was used at a concentration of 100 g/L in DMSO. PZA susceptibility tests were performed at pH 6.8 in PZA-S1 minimal medium or other media at different pH values based on the individual experiment (prepared in our laboratory) containing 0.5 g/L KH_2_PO_4_, 0.5 g/L MgSO_4_, 0.5 g/L citric acid, 10 mL/L glycerol, 2 g/L L-Ala, 30 mg/L ferric ammonium citrate, 0.5mg/L biotin, 1 mg/L pyridoxine hydrochloride and 0.5 mg/L ZnSO_4_. The pH of the medium was adjusted with 1 M of sodium hydroxide (S8045, Sigma-Aldrich, Co., St. Louis, MO, USA) and sterilized by filtering through a 0.2 µm membrane filter. For the agar plate, Bacto agar (Catalog # 214010, BD, Sparks, MD, USA) was added at a 1.5% (*w*/*v*) concentration to the PZA-S1 minimal medium, supplemented with 0.05% (*w*/*v*) albumin and 0.02% (*w*/*v*) glucose. The pH was determined with a Mettler Toledo pH meter (Model No.: FEP20 FIVE Easy Plus, Mettler Toledo, Greifensee, Switzerland).

PZA-resistant mutant colonies of *M. tuberculosis* H37Ra grown on 7H11 agar plates at pH 5.8, containing 500 mg/L of PZA after 4 weeks of incubation at 37 °C, were picked and grown in 7H9 liquid medium to confirm the PZA resistance phenotype. The PZA resistance phenotype of PZA-resistant mutants was confirmed on 7H11 agar plates containing 500 mg/L of PZA (pH 5.8) as previously described [24]. After the PZA resistance phenotype was confirmed, the *pncA* PCR was performed as described [79]. PZA-resistant *M. tuberculosis* and wild type strains are listed in Table 4. The cultures of *M. tuberculosis* were preserved in 7H9 broth supplemented with 10% (*v*/*v*) glycerol at −70 °C.

### 4.3. Determination of MICs of PZA Using Broth Microdilution Method

The broth microdilution method was used to determine the MICs as previously described [80]. In brief, the PZA concentration ranges in the well were twofold serial dilutions arranged from 800 mg/L to 12.5 mg/L or 1000 mg/L to 1 mg/L. A set of media with and without PZA control was included in each experiment. More than six colonies of *M. tuberculosis* H37Ra or PZA-resistant strains were scraped from the 7H11 agar plates using a sterile inoculating loop into a medium with 10 glass 0.5 mm beads. The sample was vortexed for 30∼60 s and settled for 15 min to remove clumps. The supernatant suspensions were diluted in the media to reach a 0.5 McFarland unit, standardized using the DEN-1B McFarland Densitometer (Grant Instruments (Cambridge) Ltd, Shepreth, Cambridgeshire, UK) according to the manufacturer’s instructions, corresponding to ∼1 × 10^7^ CFU/mL, and then diluted 1:50 and 1:100 with the PZA-S1 minimal medium used for PZA susceptibility testing via the broth microdilution method [80]. The standard inoculum size in each well was ∼1 × 10^5^ CFU/mL to ∼5 × 10^5^ CFU/mL. There is a good concordance between the McFarland scale and the CFU/mL for *M. tuberculosis* [81]. There was 0.2 mL of the bacterial suspension per well. The microtiter plates were sealed with parafilm to avoid drying and were incubated at 37 °C. The MICs—the lowest concentrations of the drug inhibiting visible growth—in liquid media were read at one, two and four weeks. The MIC results recorded by the first reader were considered to be the test results. All tests were carried out three times.

### 4.4. Determination of Activity of PZA at Neutral pH on Agar Plate

The disk diffusion method is a culture-based assay to determine antibiotic activity on solid media by observing the inhibition of visible growth [82,83]. Four-week-old colonies of *M. tuberculosis* were taken from 7H11 agar plates and suspended in PZA-S1 minimal media. The turbidity was adjusted to a 0.5 McFarland unit, standardized using the DEN-1B McFarland Densitometer (Grant Instruments (Cambridge) Ltd, Shepreth, Cambridgeshire, UK) according to the manufacturer’s instructions. In total, 5 mL of PZA-S1 agar media at 1.5% (*w*/*v*) agar was poured into each quadrant of a 100 mm Petri dish. After the agar plate solidified, a 50 µL suspension was spread on each quadrant of the Petri dish using a sterile cotton applicator. After the surface of the agar plate was dry, sterile Taxo Blank Discs (Catalog # 231039, BD, Sparks, MD, USA) with 0 (DMSO control), 400 µg, 800 µg or 1600 µg of PZA were pressed down at the center of the quadrant on the agar plate. After being sealed in plastic bags, the plates were inverted and placed in an incubator at 37 °C for 3–4 weeks. In this study, the activity of PZA was indicated by observing the inhibition of the visible growth of *M. tuberculosis* at a neutral pH at 37 °C on the agar plate. All tests were carried out three times.

### 4.5. Determination of the pH of Cultures during Growth of M. tuberculosis in PZA-S1 Media

The pH in PZA-S1 media during growth was determined colorimetrically using phenol red (3H-2,1-benzoxathiole 1,1-dioxide) (P5530, Sigma-Aldrich Co., St. Louis, MO, USA) as the pH indicator. The concentration of phenol red in PZA-S1 minimal media was 15 mg/L. In addition, the pH was checked throughout using pH paper (PANPEHA Plus pH 0–14, Whatman, GE, Marlborough, MA, USA) according to the manufacturer’s instructions. Media at serial pH values from 6.0 to 8.0 containing 15 mg/L of phenol red were used as the standard, and the cultures and control were compared with the standard to obtain the pH range [50]. All tests were carried out three times.

## 5. Conclusions

In summary, this study demonstrated, for the first time, that PZA inhibited the visible growth of *M. tuberculosis* at a neutral pH of 6.8 in PZA-S1 liquid and on an agar plate at 37 °C. It could therefore be feasible to determine the MIC of PZA to *M. tuberculosis* using a standard protocol of susceptibility testing for other TB drugs such as INH or RIF [80,84] at pH 6.8 in PZA-S1 minimal media at 37 °C. We are currently seeking cooperation to detect the susceptibility of clinical isolates to PZA and will compare this with the existing methods. This will be the subject of a future study. A reliable PZA susceptibility test is likely to have an impact on the clinical outcomes of TB treatment and the prevention of the spread of drug resistance. If implemented widely, this will have huge public health benefits.

## 6. Patents

Wanliang Shi is the founder of PZA innovation LLC and there is a patent application pending related to the invention.

## Figures and Tables

**Table 1:** MICs of PZA and growth of *M. tuberculosis* were determined at pH 6.8 in different minimal media at 37 °C for 20 days with the microdilution broth method.

Nitrogen Source and Other Ingredients ^@^	Concentration (g/L)	MIC of PZA (mg/L)	Status of Growth ^c^
L-Aspartic acid (L-Asp)	2.0	>1000	+++
L-Glutamic acid (L-Glu)	2.0	>1000	+++
L-Asparagine (L-Asn)	2.0	>1000	+++
	0.5	>1000	+++
	0.1	>1000	+++
L-Glutamine (L-Gln)	2.0	>1000	+++
L-Cysteine (L-Cys)	2.0	>1000	++
L-Histidine (L-His)	2.0	500	++
L-Proline (L-Pro)	2.0	500	++
L-Valine (L-Val) *	2.0	500	+++
L-Leucine (L-Leu)	2.0	500	++
L-Arginine (L-Arg)	2.0	250	+
L-Lysine (L-Lys)	2.0	250	+
L-Serine (L-Ser)	2.0	250	+
L-Threonine (L-Thr)	2.0	250	+
L-Isoleucine (L-Ile)	2.0	250	++
Glycine (Gly)	2.0	250	+++
L-Alanine (L-Ala)	4.0	125	+++
	2.0	125	+++
	1.0	125	+++
	0.5	125	+++
L-Methionine (L-Met)	2.0	ND	-
L-Phenylalanine (L-Phe)	2.0	ND	-
L-Tryptophan (L-Trp)	2.0	ND	-
L-Tyrosine * (L-Tyr)	2.0	ND	-

^@^ 0.5 g/L KH_2_PO_4_, 0.5 g/L MgSO_4_, 0.5 g/L citric acid, 10 mL/L glycerol, 30 mg/L ferric ammonium citrate, 0.5 mg/L biotin, 1 mg/L pyridoxine hydrochloride, 0.5 mg/L ZnSO_4_; ND, not detected. * Removing the insoluble part before adjusting the medium pH. ^c^ The amount of growth in the medium without PZA (the first column of each sample in Figure 1) could be estimated from the visible density of the *M. tuberculosis* suspension (“-” no visible growth; “+” visible growth; “++” moderate growth; “+++”, luxuriant growth).

**Table 2:** Activity of PZA against *M. tuberculosis* H37Ra according to the acidity of the PZA-S1 medium.

pH	Visible Bacterial Growth with PZA (mg/L)							
	0	12.5	25	50	100	200	400	800
8.5 *	+	+	+	+	-	-	-	-
8.0	+	+	+	+	+	-	-	-
7.5	+	+	+	+	+	-	-	-
7.0	+	+	+	+	+	-	-	-
6.5	+	+	+	+	+	-	-	-
6.0	+	+	+	+	-	-	-	-
5.5 *	+	+	-	-	-	-	-	-
5.0	-	-	-	-	-	-	-	-

* visible growth of the 4-week culture; “+”, visible growth; “-”, no visible growth.

**Table 3:** Determination of pH in the PZA-S1 medium during the growth of *M. tuberculosis*.

	Day of Incubation at 37 °C				
	0	9	15	18	30
pH of culture	6.8	~6.9	~7.0	~7.2	~6.5
pH of control *	6.8	6.8	6.8	6.8	6.8

* uninoculated media.

**Table 4:** Descriptions of the tested strains.

*M. tuberculosis* Strain	PZA Susceptibility *	*pncA* Mutation	PncA Amino Acid Substitution
*M. tuberculosis* H37Ra (ATCC 25177)	S	Wild type	
*M. tuberculosis* H37Ra (NR-122)	S	Wild type	
*M. tuberculosis* H37Ra PZA^R^ #6	R	T476C	L159P
*M. tuberculosis* H37Ra PZA^R^ #78	R	T412C	C138R
*M. tuberculosis* H37Ra PZA^R^ #98	R	T202C	W68R
*M. tuberculosis* H37Ra PZA^R^ #106	R	A146G	D49G
*M. tuberculosis* H37Ra PZA^R^ #186	R	G395C	G132A

* S, susceptible; R, resistant.

## Data Availability

The data presented in this study are available on request.
